# Profile of fasting blood glucose in obese children with insulin resistance

**DOI:** 10.1186/1687-9856-2013-S1-P100

**Published:** 2013-10-03

**Authors:** Yuri, Yose M Pangestu, Vivekenanda Pateda, Kristellina Tirtamulya, Sarah M Salendu Warouw

**Affiliations:** 1 Department of Child Health, Faculty of Medicine, University of Sam Ratulangi / RSU Prof. R. D. Kandou, Manado - Indonesia

**Keywords:** insulin resistance, fasting blood glucose level, obese children

## Background

There are more obese children in school age. They are at risk for developing type 2 diabetes in future. Fasting blood glucose level increased in obese children who have insulin resistance. We examine are there any significant differences in fasting blood glucose level of obese children among a group with insulin resistance than obese children without insulin resistance.

## Objective

To determine the difference between fasting blood glucose level in obese children with insulin resistance and without insulin resistance.

## Method

Conducted research using observational analytic research method with cross sectional approach.

## Results

There were 54 obese children, mean age 12.2 (11.9 to 12.5) years. Thirty seven (68.5%) boys and 17 (31.5%) girls. With 34 children (63%) had insulin resistance: 23 (67.6%) male sex, and 11 (32.4%) girls, 18 (52.9%) no history of diabetes mellitus in families and 16 (47.1%) were found in the family history of diabetes mellitus. The average fasting blood glucose level on average obese children with insulin resistance is higher than obese children without insulin resistance at 5.08 (4.9 to 5.2) mmol / L compared with 4.79 (4.6 to 4, 9), (p <0.003) mmol / L. Fasting Blood Glucose Level in obese children with insulin resistance was significantly higher than obese children who did not have insulin resistance.

## Conclusion

The data from this study showed that the level of fasting blood glucose in obese children who had insulin resistance is higher than obese children without insulin resistance.

